# Lung lymphatic thrombosis and dysfunction caused by cigarette smoke exposure precedes emphysema in mice

**DOI:** 10.1038/s41598-022-08617-y

**Published:** 2022-03-23

**Authors:** Barbara D. Summers, Kihwan Kim, Cristina C. Clement, Zohaib Khan, Sangeetha Thangaswamy, Jacob McCright, Katharina Maisel, Sofia Zamora, Stephanie Quintero, Alexandra C. Racanelli, David Redmond, Jeanine D’Armiento, Jisheng Yang, Amy Kuang, Laurel Monticelli, Mark L. Kahn, Augustine M. K. Choi, Laura Santambrogio, Hasina Outtz Reed

**Affiliations:** 1 Department of Medicine, Weill Cornell Medicine, New York, NY USA; 2 Department of Radiation Oncology and Caryl and Israel Englander Institute for Precision Medicine, Weill Cornell Medicine, New York, NY USA; 3 Fischell Department of Bioengineering, University of Maryland, College Park, MD USA; 4 Ansary Stem Cell Institute, Division of Regenerative Medicine, Department of Medicine, Weill Cornell Medicine, New York, USA; 5 Department of Medicine in Anesthesiology, Columbia University, New York, NY USA; 6 Department of Medicine, University of Pennsylvania, Philadelphia, PA USA; 7 Division of Pulmonary and Critical Care Medicine, Department of Medicine, Weill Cornell Medicine, 1300 York Ave, Room 323, New York, NY 10065 USA

**Keywords:** Chronic obstructive pulmonary disease, Lymphatic system, Lymphatic vessels, Experimental models of disease

## Abstract

The lymphatic vasculature is critical for lung function, but defects in lymphatic function in the pathogenesis of lung disease is understudied. In mice, lymphatic dysfunction alone is sufficient to cause lung injury that resembles human emphysema. Whether lymphatic function is disrupted in cigarette smoke (CS)-induced emphysema is unknown. In this study, we investigated the effect of CS on lung lymphatic function. Analysis of human lung tissue revealed significant lung lymphatic thrombosis in patients with emphysema compared to control smokers that increased with disease severity. In a mouse model, CS exposure led to lung lymphatic thrombosis, decreased lymphatic drainage, and impaired leukocyte trafficking that all preceded the development of emphysema. Proteomic analysis demonstrated an increased abundance of coagulation factors in the lymph draining from the lungs of CS-exposed mice compared to control mice. In addition, in vitro assays demonstrated a direct effect of CS on lymphatic endothelial cell integrity. These data show that CS exposure results in lung lymphatic dysfunction and a shift in thoracic lymph towards a prothrombic state. Furthermore, our data suggest that lymphatic dysfunction is due to effects of CS on the lymphatic vasculature that precede emphysema. These studies demonstrate a novel component of CS-induced lung injury that occurs early in the pathogenesis of emphysema.

## Introduction

Chronic Obstructive Pulmonary Disease (COPD) includes emphysema and chronic bronchitis and is commonly caused by cigarette smoke (CS). Despite extensive knowledge about the pathologic changes in the lung epithelium, blood endothelium, and the cellular mechanisms for lung injury in the pathogenesis of COPD, the lung lymphatic vasculature has not been well evaluated. The lung lymphatics drain fluid and traffic immune cells in the form of lymph from the lung parenchyma to the draining lymph nodes^1,2^. Though previously thought to be a passive conduit for lymph, an active role for the lymphatics in the inflammatory response has been increasingly the subject of investigation. Accordingly, defects in lymphatic function may play a role in the pathogenesis of disease, especially in the lungs, which are particularly dependent on lymphatic function due to the vulnerability of this organ to edema and its constant exposure to pathogens^3^.

We have previously shown that mice with lymphatic dysfunction develop lung tertiary lymphoid organs (TLOs) and lung injury with many features of human emphysema including hypoxia, breakdown of elastin, and increased MMP-12 expression^4^. TLOs are intricately associated with lymphatic vessels and resemble lymph nodes in their cellular organization and structure^5^. They are a common occurrence in lung injury and inflammation, including in COPD, where they may also be associated with disease severity^5–11^. Though lymphatic dysfunction is sufficient to cause TLO formation and emphysema in mice, it is not yet clear whether TLOs that are seen in emphysema due to CS are associated with lymphatic dysfunction.

In this study, we sought to uncover the effect of CS on the lung lymphatic vasculature. We found that lung lymphatic vessel thrombosis is present in lung tissue from patients with emphysema due to CS exposure, and that this increases with disease severity. Using a mouse model, we found that CS caused lung lymphatic thrombosis and dysfunction as well as changes in the composition of thoracic lymph, and that this occurred prior to the development of emphysema. In addition, cigarette smoke extract had a direct effect of lymphatic endothelial cell integrity.

## Results

### Increased expression of the lymphatic marker *PROX1* in COPD lung tissue

**Figure 1:**
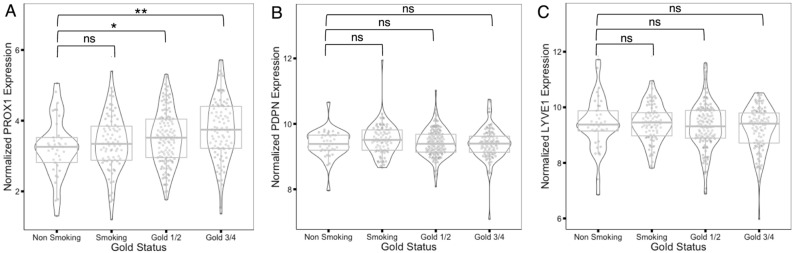
Expression of lymphatic endothelial cell markers in human COPD lung tissue*. *In silico analyses of microarray datasets from the LTRC tissue consortium for *PROX1* (**A**) Podoplanin (*PDPN*, **B**), and *LYVE1* (**C**) in lung tissue from nonsmoker control, smoker control, GOLD1/2 (mild COPD), and GOLD3/4 (severe COPD) lungs. *P < 0.05, **P < 0.01.

Previous studies have used immunohistochemistry to show an increase in lymphatic markers in lung tissue from patients with COPD^12,13^. We extended these studies and used microarray datasets from the Lung Tissue Research Consortium (LTRC) to quantify expression of lymphatic markers in lung tissue from patients with COPD. We found an upregulation in the expression of the essential lymphatic transcription factor *PROX1* that increased with disease severity (Fig. 1A). Interestingly, we did not find a change in the expression of *Podoplanin* (*PDPN*) or *LYVE1* (Fig. 1B,C), which may reflect decreased specificity of these genes for the lymphatic endothelium^14,15^, or differences in the technique used to analyze the tissue compared to previous studies.

### Lung lymphatic thrombosis in severe emphysema

**Figure 2:**
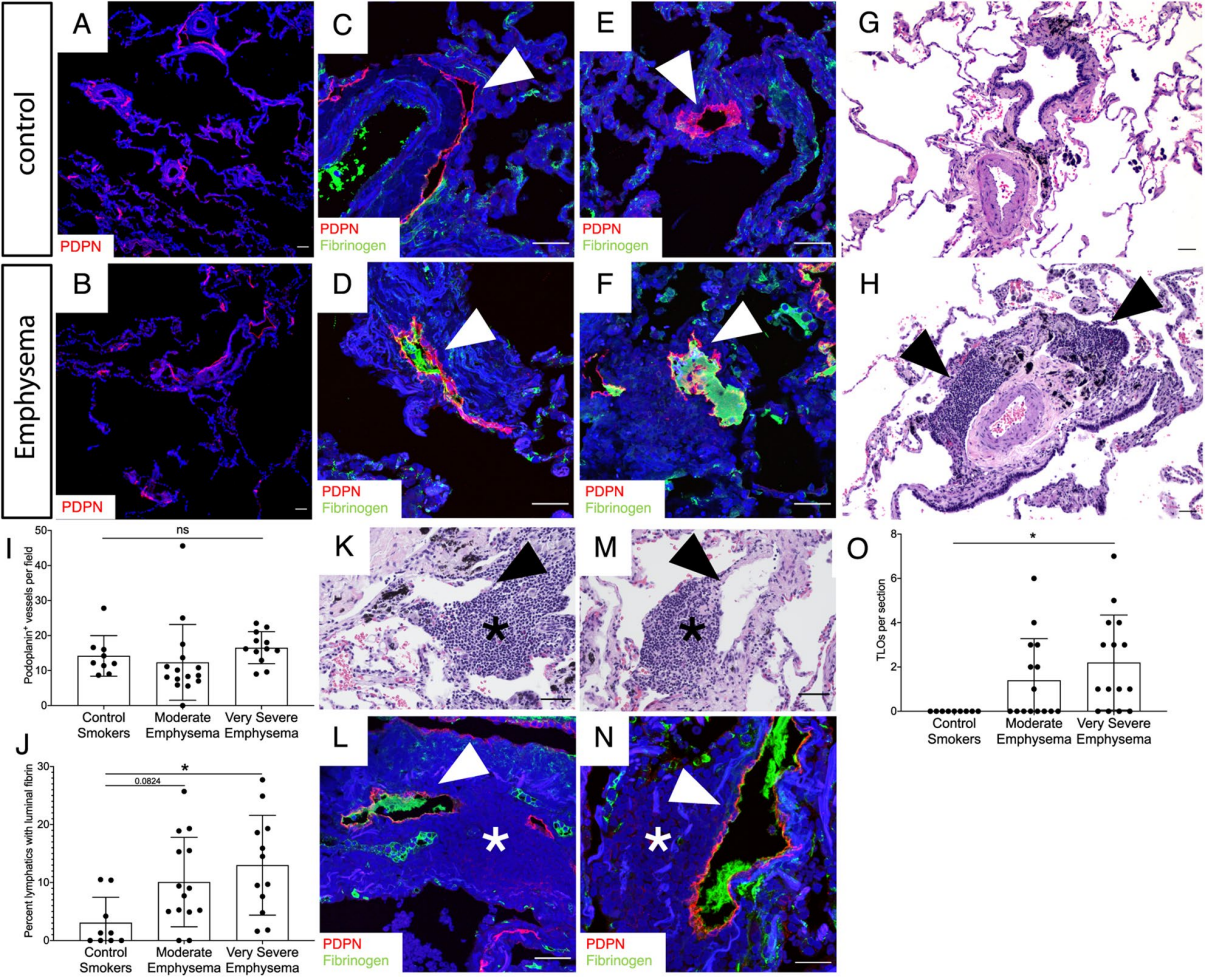
Lymphatic thrombosis in human emphysema. (**A**,**B**) Representative fluorescent immunohistochemical analysis of lung tissue from patients with emphysema or control smokers for the lymphatic marker PDPN (red). (**C**–**F**) Immunohistochemical staining for lymphatic thrombosis using PDPN (red) and fibrinogen (green) in lung tissue from patients with emphysema (**D**,**F**) and control smokers (**C**,**E**). Lymphatics (red) are indicated by arrowheads. (**G**,**H**) H&E staining of lung tissue revealed TLOs in emphysema (**H**, arrowheads) but not control lung tissue (**G**). (**I**) Quantification of PDPN^+^ lung lymphatics in tissue from control smokers and patients with moderate and very severe emphysema. (**J**) Quantification of PDPN^+^ lymphatic vessels with luminal fibrin. (**K**–**N**) Serial H&E and immunohistochemistry serial sections (**K**,**L** and **M**,**N**) of lung tissue from patients with very severe emphysema demonstrating thrombosed lymphatics (arrowheads) spatially associated with TLOs (asterisks). (**O**) Quantification of TLOs in human lung tissue. Quantification lung lymphatic vessels was performed by counting PDPN^+^ lymphatics in 10 × images of lung tissue sections. At least 3 10 × images were used for each patient tissue sample and the average lymphatic number was determined. Lymphatic thrombosis was quantified as the percentage of PDPN^+^ lung lymphatics with luminal fibrin in each 10 × image and was averaged from at least 3 10 × images. TLOs were quantified as the total number of TLOs visualized in each lung tissue sample using H&E staining. All values are means ± SEM. *P* value calculated by ANOVA. *P < 0.05. *ns* not significant. Scale bars = 25 μm.

While our microarray data suggested increased expression of the lymphatic marker *PROX1* in COPD, this did not address possible changes in the morphology or function of these vessels. We analyzed the lymphatics in lung tissue from patients with a history of cigarette smoking that have been and clinically and radiographically identified as having emphysema by the LTRC. Using immunohistochemical analysis for PDPN, we found no change in the density of lung lymphatics in patients with emphysema compared to control smokers, in agreement with our microarray data (Fig. 2A,B,I). However, careful analysis revealed fibrin-rich thrombi in the lymphatics of patients with emphysema, in some cases obstructing the entire lumen of the vessel (Fig. 2C–F). Lymphatic thrombosis was significantly increased in patients with very severe emphysema (GOLD IV) compared to moderate emphysema (GOLD II) or control smokers (Fig. 2J). TLOs were similarly increased in very severe emphysema (Fig. 2G,H,O), as seen in previous studies^6^. Interestingly, we identified thrombosed lymphatic vessels that were spatially associated with TLOs in some samples (Fig. 2K–N).

### Lung lymphatic thrombosis after cigarette smoke exposure in mice

**Figure 3:**
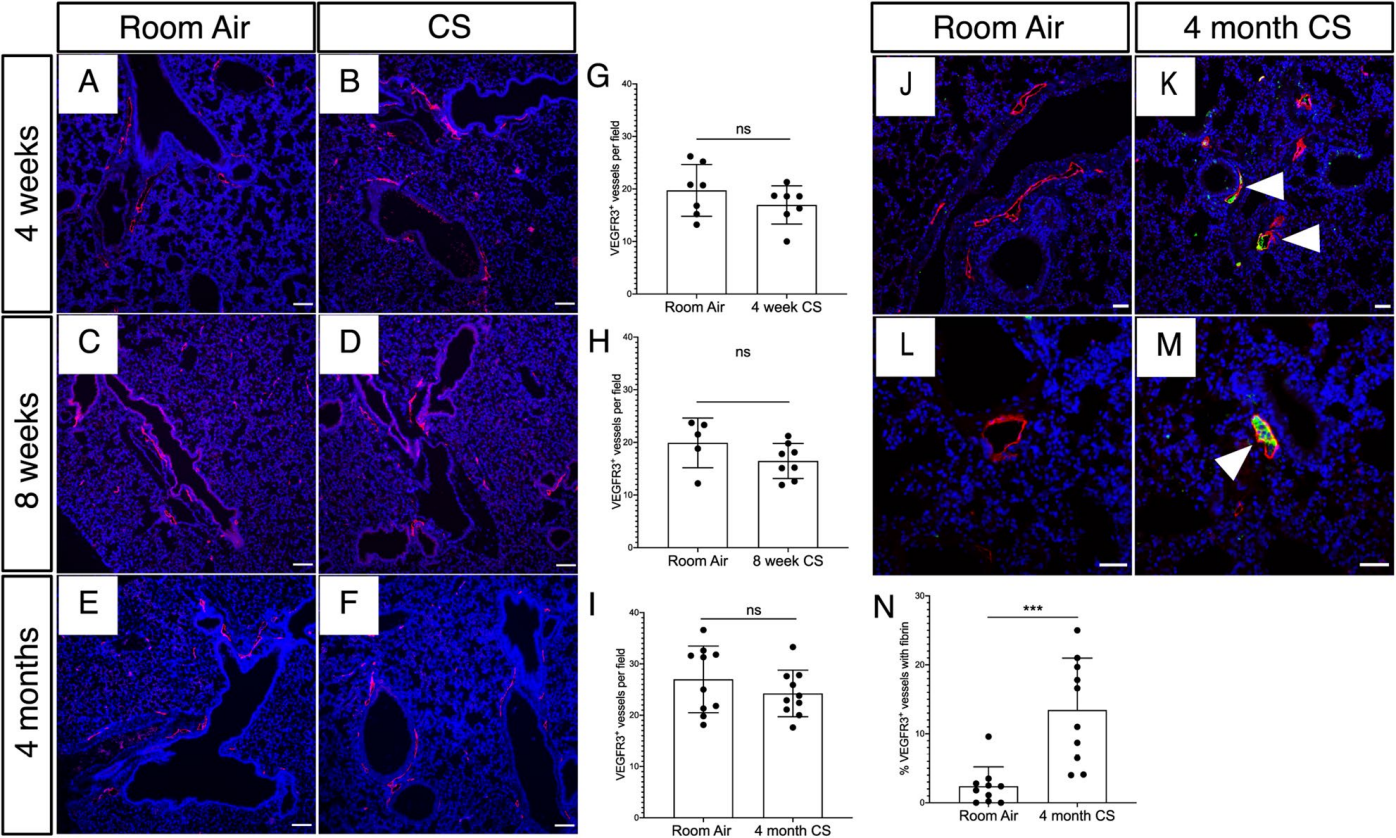
Lymphatic thrombosis after CS exposure in mice. Mice were exposed to full body CS and the lungs were harvested for fluorescent immunohistochemical analysis. (**A**–**F**) Representative images of immunohistochemical staining of lung tissue for the mouse lymphatic marker VEGFR3 (red) in mice exposed to CS for 4 weeks, 8 weeks, and 4 months compared to identically housed age matched mice exposed to room air. (**G**–**I**) Quantification of lung lymphatic vessel density in CS-exposed and control mice at the indicated time points. (**J**–**M**) Analysis of lung lymphatic thrombosis using immunohistochemical staining for VEGFR3^+^ (red) and fibrinogen (green). Thrombosed lymphatics indicated with arrowheads. (**N**) Quantification of lymphatic thrombosis, as expressed by percentage of VEGFR3^+^ vessels with luminal fibrin after 4 months of CS exposure. Quantification lung lymphatic vessels was performed by counting VEGFR3^+^ lymphatics in 10 × images of lung tissue sections. At least 5 10 × images were used for each tissue sample and the average lymphatic number was determined. Lymphatic thrombosis was quantified as the percentage of VEGFR3^+^ lung lymphatics with luminal fibrin in each 10 × image and was averaged from at least 3 10 × images. ***P < 0.001. *ns* not significant. Scale bars in (**A**–**F**,**J**,**K**) = 25 μm. Scale bars in (**L**,**M**) = 50 μm.

Our analysis of human samples from patients with emphysema could not elucidate whether lymphatic thrombosis occurs prior to emphysema and is a result of CS exposure or whether it is secondary to lung remodeling in this disease. We sought to investigate whether CS affects lung lymphatic function prior to emphysema using a whole body exposure system in mice^16^. Using immunochemistry for the lung lymphatic marker VEGFR3^4,14^, we found no difference in lung lymphatic density after 4 weeks, 8 weeks, or 4 months of smoke exposure compared to identically housed control mice at the same time points (Fig. 3A–I). However, further analysis revealed fibrin-rich clots in the lymphatics of CS-exposed mice that were not seen in control mice, and in some cases completely obstructed the lumen of the vessel, similar to what we observed in human emphysema (Fig. 3J–N). Lymphatic thrombosis was seen within 4 months of CS exposure, prior to the development of emphysema in this model (Supplemental Fig. 1A), suggesting that tissue remodeling was not the major driver of thrombosis in these vessels. We rarely observed TLOs in our samples at this timepoint (Supplemental Fig. 1B), in agreement with previous reports that TLOs are most consistently observed after 6 months or more of CS exposure, with the onset of emphysema^17,18^. However, we identified thrombosed lymphatic vessels that were spatially associated with the sporadic TLOs that were present in the lungs of CS-exposed mice at this timepoint, similar to what we observed in human emphysema (Supplemental Fig. 1C–H). To confirm that lymphatic thrombosis was not a result of emphysema, we used the elastase model, in which tracheal instillation of porcine pancreatic elastase results in severe lung injury and progressive alveolar breakdown resulting in emphysema within 21 days^19^. Elastase treatment did not affect lung lymphatic density, and we did not observe any significant lymphatic thrombosis in this model, despite severe emphysema and alveolar damage (Supplemental Fig. 2A–F). Interestingly, elastase treatment also did not result in TLO formation in association with the development of emphysema, even in areas with most significant alveolar enlargement and tissue destruction (Supplemental Fig. 2G,H). These data suggest that lung lymphatic thrombosis is caused by CS exposure and not from lung remodeling or emphysema.

### Cigarette smoke exposure leads to decreased lung lymphatic function

**Figure 4:**
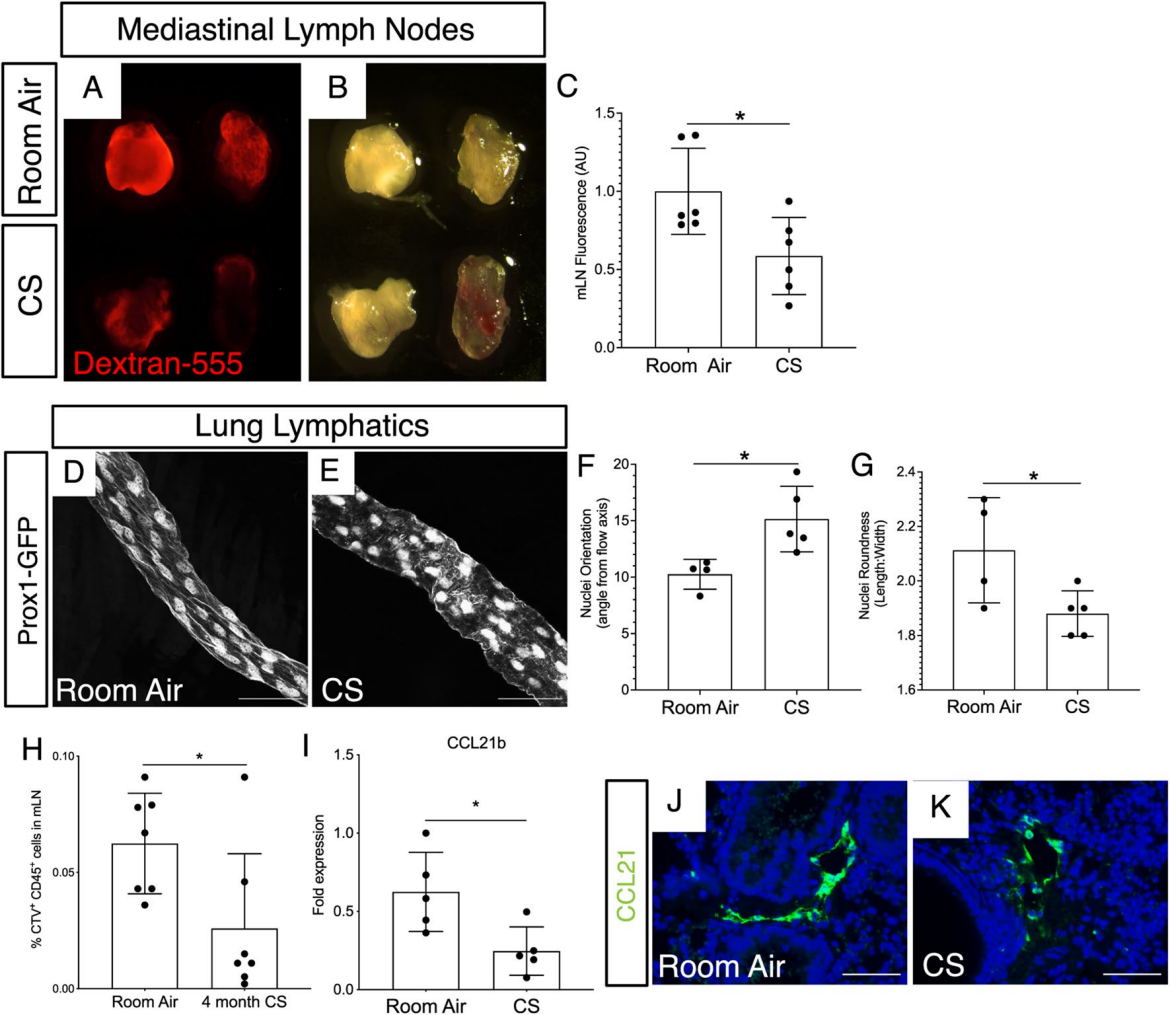
Lung lymphatic dysfunction after CS exposure in mice. Fluorescence (**A**) and brightfield (**B**) microscopy of mediastinal lymph nodes (mLN) from mice after 4 weeks CS exposure or room air. mLNs were harvested 60 min after intra-tracheal administration of dextran-555 (red). (**C**) Quantification of dextran-555 uptake to mediastinal lymph nodes by mean fluorescence intensity (AU). (**D**,**E**) Whole mount microscopy of lung lymphatics from *Prox1-EGFP* mice exposed to CS (**E**) or room air (**D**) for 8 weeks. (**F**,**G**) Quantification of nuclear orientation (angle from vessel axis) and nuclear roundness (length:width ratio) in lung lymphatics after 8 weeks CS or room air. (**H**) Cell trafficking assay to assess lymphatic leukocyte migration from lungs to draining lymph nodes was performed using intra-tracheal administration of CTV-labeled leukocytes followed by harvest of mLNs for flow cytometry. Quantification of CTV^+^ leukocytes in mLNs is shown as percent of total cells by flow cytometry. (**I**) Expression of CCL21b by quantitative PCR using whole lung tissue from mice after 4 months of CS exposure compared to room air, normalized to GAPDH. (**J**,**K**) Immunohistochemistry for CCL21 (green) using lung tissue from mice after 4 month CS or room air. Whole mount microscopy images are representative of lung tissue from at least 4 mice in each group. All values are means ± SEM. *P* value calculated by Student’s *t* test. *P < 0.05. *ns* not significant. Images in (**A**,**B**) shown at 5 × magnification. Scale bars in (**D**,**E**,**J**,**K**) = 50 μm.

We next asked if the finding of lung lymphatic thrombosis was associated with decreased drainage in these vessels in CS-exposed mice prior to emphysema. To do this, we used a dextran drainage assay, in which fluorescently labeled dextran is delivered to mice intratracheally, and lung lymphatic drainage is quantified by detection of the fluorophore in the mediastinal lymph nodes (mLNs)^4^. Dextran drainage from the lungs to mLNs was significantly decreased in mice after 4 weeks of CS exposure, as assessed by both visualization of the mLNs and quantification of mLN fluorescence (Fig. 4A–C). Changes in vascular flow are often reflected in endothelial cell morphology, with increased nuclear roundness and changes in nuclear orientation being a hallmark of impaired flow^20^. We used whole mount microscopy in *Prox1-EGFP* lymphatic reporter mice, in which lymphatic endothelial cells express GFP^21^, to further assess the lung lymphatics in CS-exposed mice after 8 weeks. We found that lung lymphatic vessels in CS-exposed mice have an altered morphology compared to lung lymphatics in control mice, with rounded lymphatic endothelial cell nuclei that deviated from the axis of flow (Fig. 4D,E). These changes were confirmed by quantification of nuclear orientation and length to width ratio (Fig. 4F,G).

A major role of the lung lymphatics is to facilitate migration of immune cells from the lungs to the draining mediastinal lymph nodes^4,22–24^. We tested the effect of CS on lung lymphatic leukocyte trafficking. Leukocytes labelled with cell trace violet (CTV), a fluorescent dye, were administered intratracheally to mice, and their presence in the draining mLNs was detected by flow cytometry^4^. We found significantly decreased lung lymphatic leukocyte trafficking in CS-exposed mice after 4 months compared to control mice (Fig. 4H). Furthermore, expression of CCL21b, a cytokine that is expressed by the lung lymphatic capillaries and is critical for leukocyte uptake and migration^25^ was decreased in CS-exposed mice by quantitative PCR and immunohistochemical analysis (Fig. 4I–K). Interestingly, expression of CCL21a, an isoform in mice that is not specific for the lymphatics and is more broadly expressed in secondary lymphoid tissue^26^ was unchanged in CS-exposed mice by quantitative PCR (data not shown). Taken together, these findings indicate decreased lung lymphatic function in CS-exposed mice with thrombosis, decreased drainage, and impaired leukocyte trafficking in these vessels at timepoints that precede emphysema.

### CS exposure alters the composition of lymph towards a prothrombotic and inflammatory state

**Figure 5:**
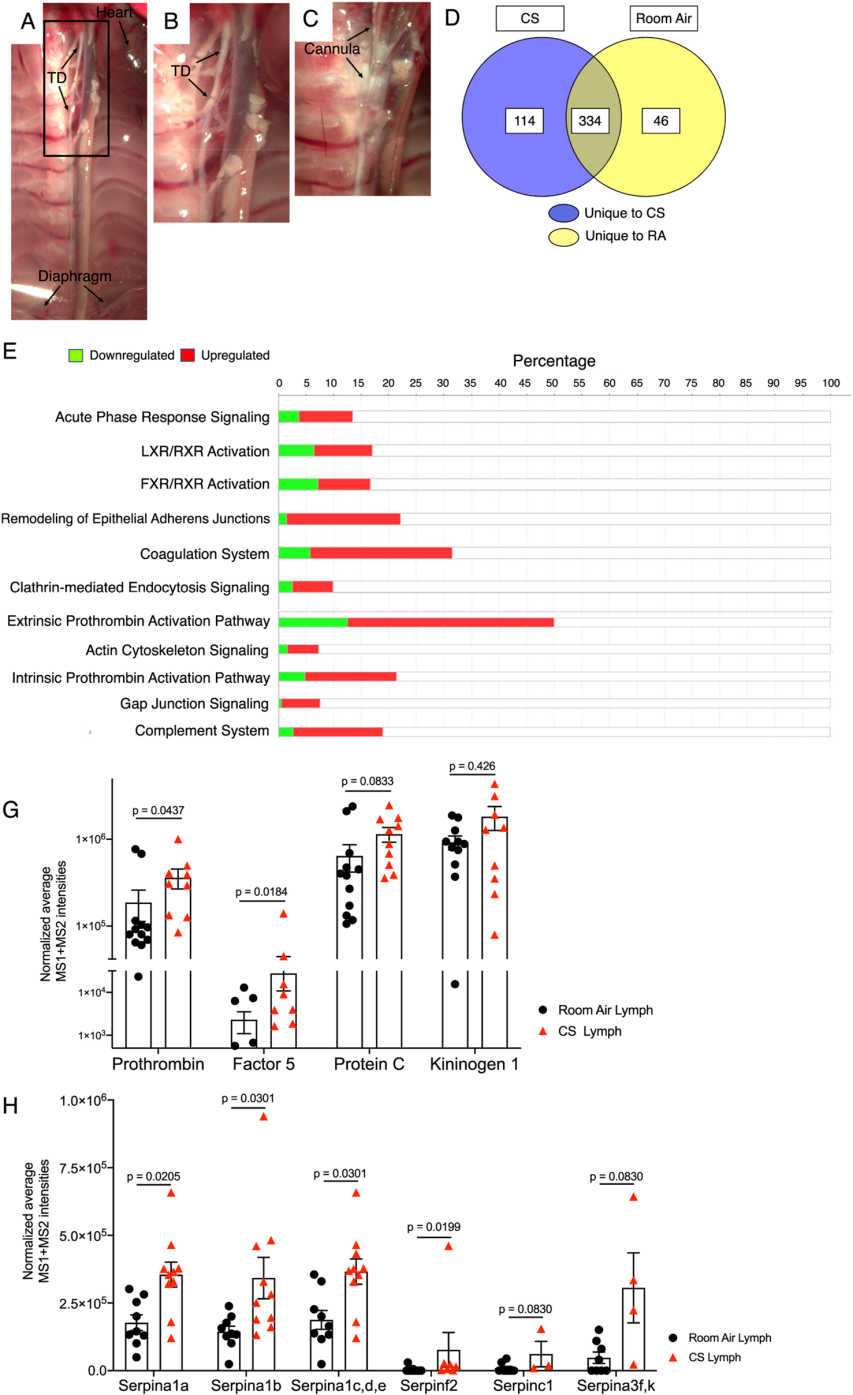
Proteomic analysis of thoracic lymph from CS-exposed mice. (**A**) Brightfield microscopy demonstrating the thoracic duct (TD) in mice relative to other structures. (**B**,**C**) Higher magnification image of region indicated in (**A**) showing harvest of lymph via cannulation of TD (arrows). (**D**) Label free proteomic analysis of lymph after 8 weeks of CS exposure identified 114 unique proteins in lymph from CS-exposed mice and 46 unique proteins in lymph from room air mice. 334 overlapping proteins were seen in these groups, of which 151 were upregulated and 107 were downregulated in lymph from CS-exposed mice compared to room air mice. The proteomics data set are presented in Supplementary Table 1. (**E**) Ingenuity pathway analysis (IPA) of DIA proteomics data set presented in the Supplementary Table 1 retrieved top biochemical pathways displaying quantitative changes in the lymph of CS-exposed mice. The experimentally determined protein ratios corresponding to [CS/Room Air] from MS1 and MS2 analysis of DIA proteomics data were used to calculate the experimental fold changes by rescaling their values using a log2 transformation, such that positive values reflected fold increases (red color) while the negative values reflected fold decreases (green color). Results from 2 independent experiments with a total of n = 10 CS-exposed mice and n = 12 room air control mice are displayed in IPA using stacked bars while the percentage overlapped proteins from the experimental data set with the IPA knowledge data base for reach pathway are shown on the X axis. The probability of having a relationship between each IPA indexed biological function and the experimentally determined protein was calculated by right-tailed Fisher’s exact test with the Benjamini–Hochberg Correction. The statistical significance was set to a P-value of < 0.05. (**G**) Relative abundance of coagulation proteins in lymph from CS-exposed or room air control mice, as quantified by normalized average MS1 + MS2 intensities from DIA proteomics data. (**H**) Relative abundance of serpine proteins in lymph from CS-exposed or room air control mice, as quantified by normalized average MS1 + MS2 intensities from DIA proteomics data. Results are reported as mean ± SDV (n = 12 for room air and n = 10 for CS samples). The statistical significance corresponding to P < 0.05 was calculated in GraphPad Prism 9 (GraphPad Software, La Jolla, CA) using the multiple nonparametric Mann–Whitney *t* tests discovery analysis with Holm–Sidak method (alpha = 0.05).

**Figure 6:**
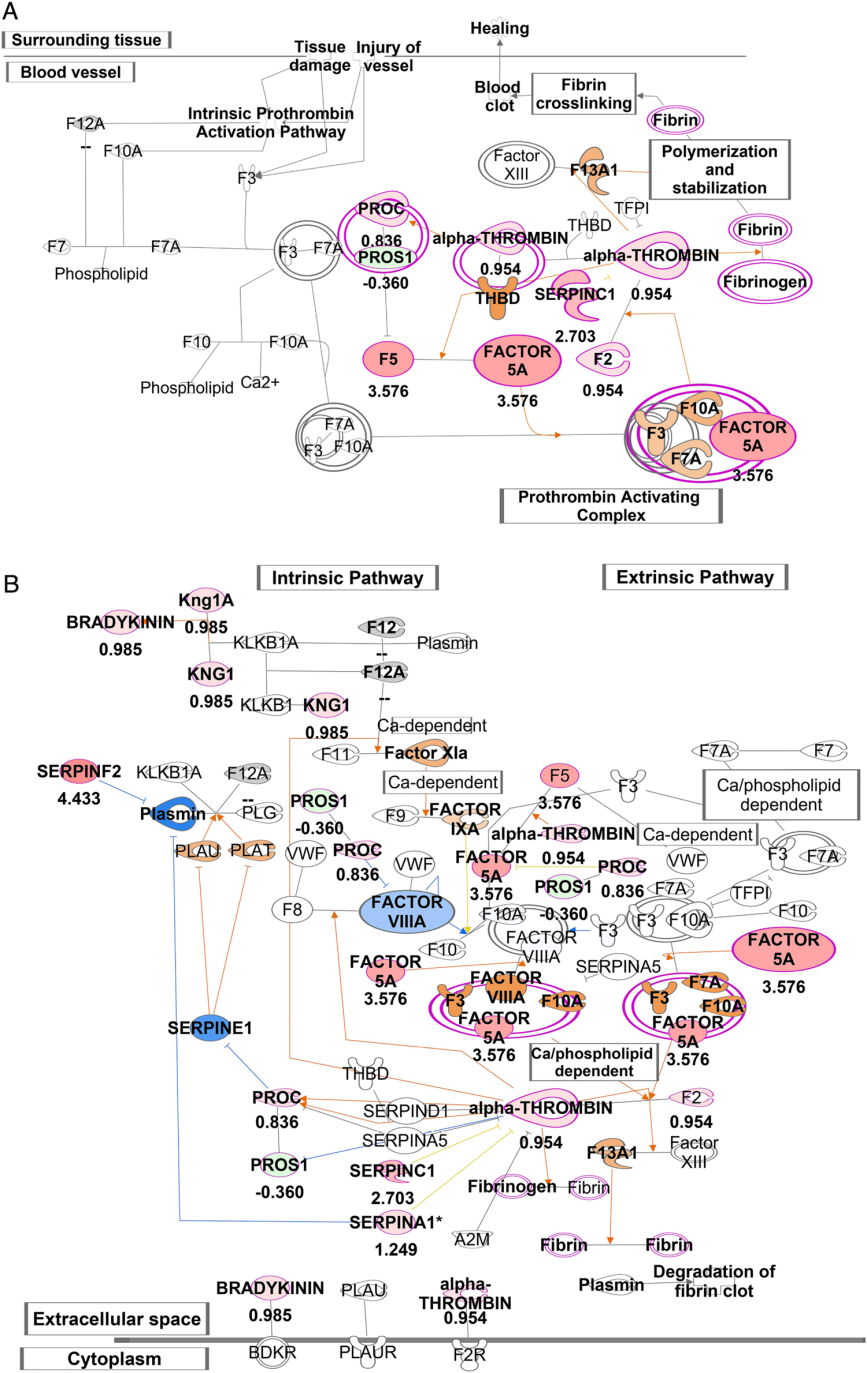
Upregulation of coagulation in thoracic lymph from CS-exposed mice. View of IPA-predicted prothrombin activation pathway (**A**) and coagulation system (**B**) in lymph from CS-exposed mice compared to room air mice. The experimental log ratio of proteins in lymph from CS-exposed versus room air mice are indicated. Experimentally determined proteins that were upregulated are depicted in red, and those that were downregulated are shown in green. Proteins that were predicted by IPA to be inhibited due to their networking relationship with the experimentally determined proteins are shown in blue, and proteins that were predicted to be activated are shown orange. IPA predicted enhanced coagulation in the lymph from mice exposed to smoke as compared with room air, mainly due to the increased in the abundance of Factors 5/5A, alpha-thrombin, SERPINSF2, C1, A1 and Kininogen 1 (shown in red). Accordingly, IPA predicted an increased in the activation of other coagulation factors like FVIIIa, F3, F10AF7a, FXIa, FIXa and F13A1 (orange). IPA also predicted a decrease in plasmin activity (blue). Results from 2 independent experiments with a total of n = 10 CS-exposed mice and n = 12 room air mice.

We next investigated whether decreased lung lymphatic dysfunction is associated with changes in the composition of lymph in CS-exposed mice. Lymph is a combination of interstitial fluid, products of tissue metabolism, and immune cells, and therefore reflects the physiologic and pathologic signature of the tissue it originated from^1,27,28^. We analyzed the proteomic signature of lymph from mice exposed to 8 weeks of CS compared to room air, as this is a timepoint where we have observed decreased lymphatic function but is prior to lymphatic thrombosis. Lymph was harvested from the thoracic duct at a site that is likely to be anatomically enriched for lung drainage (Fig. 5A–C). Proteomic analysis revealed a significant number of unique proteins in lymph from CS-exposed mice, as well as changes in the relative abundance of proteins that were expressed in each group (Fig. 5D, Supplemental Table 1). Pathway analysis of the proteome from lymph from CS-exposed and control mice showed upregulation of several proinflammatory pathways, including acute phase response signaling, the extrinsic and intrinsic prothrombin activation pathway, and the complement system (Fig. 5E). Upon further analysis, we detected a significant increase in several proteins involved in the coagulation cascade in the lymph from CS-exposed mice, including Prothrombin and Factor 5 (Fig. 5G). In addition, we found a significant increase in Serpin proteins, which are serine proteases that are involved in coagulation and inflammation (Fig. 5H). We also identified an increase in several proteins in the complement cascade (Supplemental Fig. 3). IPA predicted enhanced coagulation-mediated inflammation in lymph from mice exposed to smoke compared to room air, which could be attributed to the increased abundance of Factor 5A, alpha-thrombin, SERPINSF2, C1, A1 and kininogen 1 (Fig. 6A,B). As a result of these observed protein increases, IPA predicted an increase in the activation of other coagulation factors including FVIIIa, F3, F10AF7a, FXIa, FIXa and F13A1 (Fig. 6B). Furthermore, the increased abundance of SERPINF2 was predicted to trigger decreased activity of Plasmin, which SERPINF2 inhibits. Because plasmin is important for degradation of fibrin clots, the predicted model supported decreased fibrinolysis and accumulation of fibrin clots in lymph from CS-exposed mice compared to control. These data suggest that lymphatic dysfunction that we observe after smoke exposure are associated with simultaneous changes in lung lymph that promote thrombosis.

### Cigarette smoke extract causes increased lymphatic endothelial cell permeability in vitro

**Figure 7:**
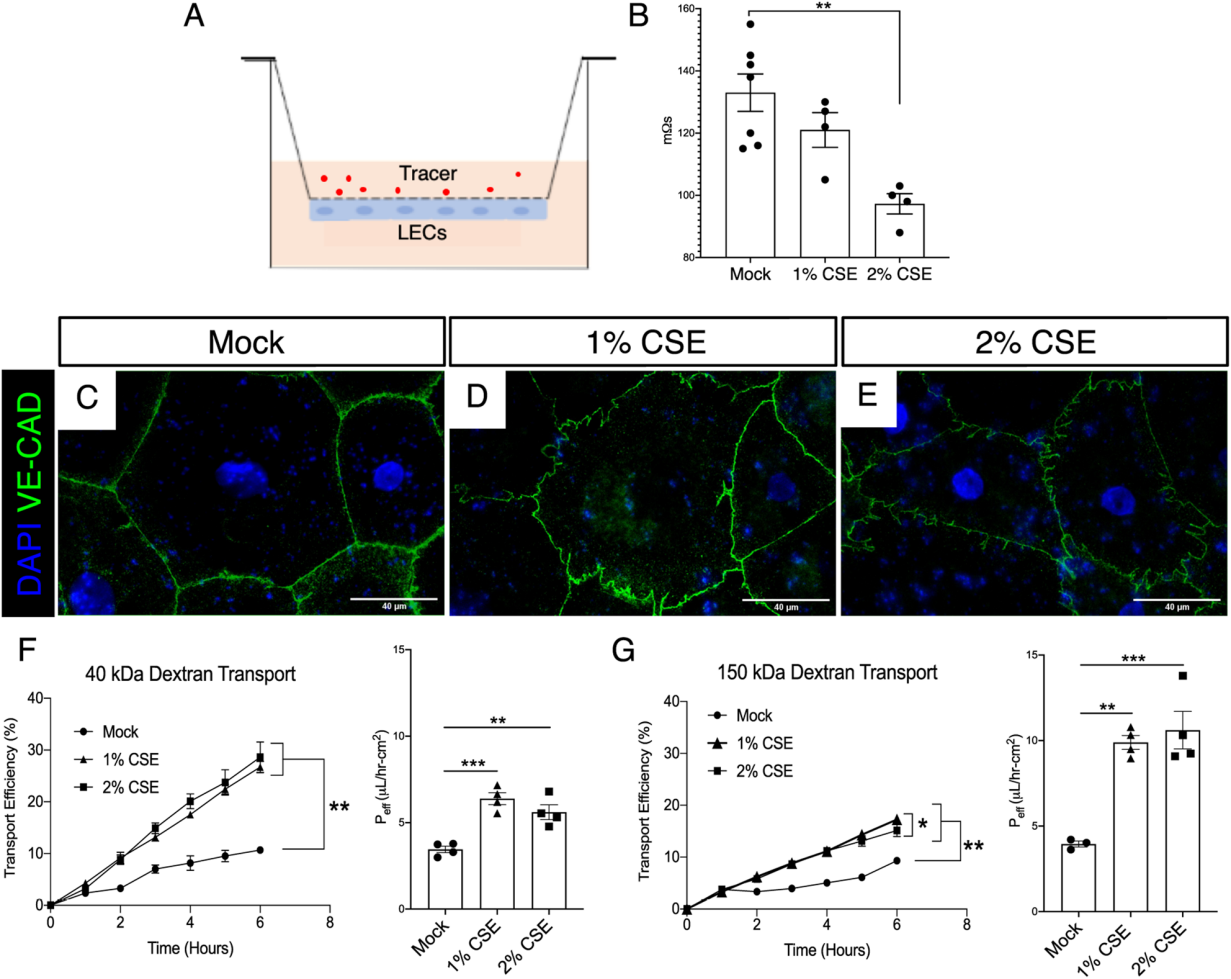
Cigarette smoke extract increases LEC permeability in vitro. (**A**) Schematic of LEC transport model where LECs are seeded on the bottom of a flask and transport of a fluorescent tracer across the monolayer is assessed. (**B**) Transendothelial electrical resistance (TEER) of a monolayer of LECs after treatment with 1% or 2% CSE for 12 h. (**C**–**E**) Representative fluorescent images of LECs stained for VE-cadherin (VE-CAD, green) after treatment with CSE for 24 h. (**F**) Transport efficiency of 40 kDa dextran across LEC monolayer shown over time (left) and as effective permeability (right, P_eff_ µl/h-cm^2^) at 6 h (n = 3–4). (**G**) Transport efficiency of 150 kDa dextran across LEC monolayer over time (left) and effective permeability (right, P_eff_ µl/h-cm^2^) at 6 h (n = 3–4). *P < 0.05, **P < 0.01, ***P < 0.001. Scale bars = 40 µm. Microscopy images are representative of at least 4 replicates per group.

Our in vivo data demonstrated that lymphatic dysfunction and a prothrombotic shift in of lymph occur prior to the development of emphysema, suggesting that CS may be directly injurious to the lymphatic endothelium. We therefore sought to determine the effect of CS on the lymphatic endothelium. To do this, we use used an in vitro endothelial cell transport model where lymphatic endothelial cells (LECs) are seeded on transwell inserts and allowed to reach confluency for 48 h before being exposed to 1 or 2% (v/v) cigarette smoke extract (CSE) (Fig. 7A). After 12 h, trans-endothelial resistance (TEER) was measured. We found that LEC TEER was decreased with CSE in a dose-dependent manner (Fig. 7B). To determine whether the reduction in TEER may be due to changes in LEC cell–cell junctions, we used immunocytochemical staining for the junctional protein VE-Cadherin, which a key protein involved in lymphatic vasculature junctions and is found in all junction types in these vessels^29,30^. We found that LEC cell–cell junctions after CSE appeared jagged and less continuous compared to control LECs (Fig. 3C–E). This was not due to changes in the expression of VE-cadherin or other junctional proteins (Supplemental Fig. 4). We next tested whether decreased resistance and junctional changes correlated with increased permeability of LECs to bio-inert solutes, specifically 40 kDa and 150 kDa dextran. We found that treatment with CSE led to increased transport of both 150 kDa Dextran and 40 kD dextran across LECs in a time dependent fashion (Fig. 7F,G). These studies reveal a direct effect of CSE on LEC permeability.

## Discussion

The lung lymphatics play an important role in lung homeostasis due to their role in fluid drainage and immune cell trafficking. Despite this, lymphatic function in the pathogenesis of chronic lung diseases such as COPD has been less rigorously investigated. Previous studies have documented changes in the density of lung lymphatic vessels in human COPD^12,13^ and our microarray studies using human tissue generally agreed with these reports. However, whether this reflects changes in lymphatic function was unknown. Furthermore, while previous studies have investigated the role of immune cell trafficking in the lungs and draining lymph nodes in a mouse model of CS-induced COPD, these studies have not directly addressed the role of the lymphatic endothelium^31,32^. Our previous work showed that lymphatic dysfunction results in TLO formation and emphysema in mice. Because TLOs are a prominent feature of CS-induced emphysema^6^, we reasoned that TLOs may be a marker of previously unidentified lymphatic dysfunction in this disease. Here, we have shown that lung lymphatic thrombosis is increased in patients with emphysema compared to control smokers. Furthermore, lung lymphatic thrombosis was increased in patients with severe emphysema compared to moderate disease. Though not necessarily indicative of impaired lymphatic function, it is reasonable that lymphatic thrombosis reflects impaired lymphatic drainage, as is the case in other settings where lymphatic thrombosis has been observed^33–35^. However, without longitudinal analysis of the human lung tissue, we could not determine from these data whether lymphatic thrombosis is predisposing factor associated with severe emphysema that occurs early in this disease, or alternatively, whether severe emphysema and tissue remodeling play a causal role in lung lymphatic thrombosis apart from CS exposure. Due to these limitations, we used mouse models of emphysema to further explore the direct effect of CS on lymphatic function.

We observed lymphatic thrombosis in CS-exposed mice which was histologically identical to what we observed in human emphysema tissue. Functional studies confirmed that lymphatic thrombosis was associated with decreased lung lymphatic drainage and morphologic changes in the lung lymphatic endothelium indicative of impaired lymph flow in mice with CS-exposure. Furthermore, lung lymphatic dysfunction after CS exposure culminated in impaired immune cell trafficking. Importantly, lymphatic dysfunction and thrombosis occurred at timepoints that preceded the development of emphysema in this model, suggesting that CS exposure, and not tissue remodeling, is the cause of lymphatic thrombosis and dysfunction. The temporal sequence of CS leading to lymphatic dysfunction prior to emphysema was further supported by the finding that severe elastase-induced emphysema alone in the absence of CS exposure was not sufficient to induce lymphatic thrombosis. Given the severe lung injury and emphysema in the elastase model, these data suggest a causal role for CS in development lung lymphatic thrombosis that is independent from emphysema itself. Taken together, our studies show that lymphatic dysfunction and thrombosis are among the initial changes that occur in the lung in response to CS exposure. Though we did not investigate lymphatic thrombosis and dysfunction after chronic CS exposure in our model, that lymphatic thrombosis is seen in tissue from patients with severe emphysema suggests that these changes may occur with smoke exposure and persist as emphysema progresses. This previously unrecognized effect of CS on lung lymphatic function suggests a novel component in the pathogenesis of emphysema.

Lymphatic thrombosis is generally rare and occurs far less frequently than thrombosis in the blood vascular system. This is because despite the presence of fibrinogen and coagulation factors, lymph is generally a hypocoagulable fluid that lacks platelets and has relatively strong fibrinolytic activity^35^. Despite this, lymph does clot in pathologic conditions, and previously reported causes of lymphatic thrombosis include cancer (typically due to external compression and subsequent stasis), infections, heart failure, or chronic edema^33–37^. To our knowledge, our studies are the first to show lymphatic thrombosis in human emphysema and in response to CS-exposure in a mouse model. CS-induced lymphatic thrombosis may reflect a similar effect of CS on the lymphatic endothelium as in the blood vascular system, where there is well-documented endothelial cell injury and coagulation abnormalities in response to CS^38–40^. Supporting direct injury of CS on the lymphatic endothelium, we found that cigarette smoke extract causes increased LEC permeability. Increased permeability in lymphatics could promote thrombosis through exposure of tissue factor and activation of the coagulation cascade in a similar manner as is seen in settings of increased blood vascular permeability. Indeed, our proteomic analysis of lymph from CS-exposed mice showed upregulation of pathways of coagulation and shifts in the relative abundance of proteins to promote clot formation. The effect of CS on the lymphatic vasculature may therefore involve both direct injury to the lymphatic endothelium as well as changes in the composition of lymph towards a prothrombotic state. It is also conceivable that activated leukocytes that traffic in the lymphatics in the setting of CS exposure may play a role in lymphatic thrombosis and initiation of the coagulation cascade. In either scenario, LEC injury and increased permeability, inflammation in the setting of impaired lymph flow, and the known prothrombotic effects of CS could fulfill the tenants of ‘Virchow’s triad’ and trigger thrombosis in these vessels. These previously unappreciated changes in lymph and lymphatic function after CS exposure prior to emphysema may also provide insights into the early pathogenesis of these disease that drives subsequent lung injury.

In this study, we have shown that lymphatic thrombosis and dysfunction occur after CS exposure in mice, and that lung lymphatic thrombosis increases with disease severity in human emphysema. Though not addressed here, our studies raise the possibility that lymphatic dysfunction may either play a role in the pathogenesis in emphysema, or be a marker of disease progression, or both. Apart from the thrombosis itself, activation of thrombin in the lymphatic endothelium may cause dysfunction through a variety of mechanisms, as is seen in the blood vasculature^41–43^. Furthermore, given the fundamental role of the lung lymphatics in leukocyte trafficking and regulation of the inflammatory response, a downstream effect of lymphatic dysfunction may be accumulation and activation of immune cells that subsequently cause tissue injury. Given that lymphatic dysfunction results in decreased lung immune cell trafficking and formation of TLOs^4^, the work presented here raises the possibility that TLOs in COPD are due at least in part to lymphatic dysfunction that precedes their formation. The mechanistic role of lymphatic dysfunction and thrombosis in TLO formation and emphysema will be the subject of future investigations.

## Materials and methods

### Human lung samples

De-identified human samples were obtained from the NHLBI Lung Tissue Research Consortium (LTRC) biorepository (https://biolincc.nhlbi.nih.gov/studies/ltrc/). These samples were obtained from donor subjects who were planning lung surgery, using tissue that would otherwise be discarded after the lung surgery. Tissue was submitted with a standardized series of questions as well as pulmonary function tests, six-minute walk tests, and chest imaging. For microarray analysis, we analyzed specimens from patients with severe COPD (GOLD 3/4), mild COPD (GOLD 1/2), no COPD (Smoker control), and non-smoker controls. Normalized gene expression profiling of chronic lung disease for the Lung Genomics Research Consortium was downloaded from the Gene Expression Omnibus, under accession number GSE47460 and as reported previously^44^. After analyzing clinical metadata, generalized linear models were created for select gene expression, GOLD Status and the microarray platform using the glm function in the R stats package. Boxplots and scatterplots were made using ggplot2 R package. Generalized linear model Wald statistics were used to assess significance of coefficients in relevant models. For immunohistochemical analysis, we further restricted our analysis to tissue from patients that were identified by the LTRC as having pathologic and radiographic evidence of emphysema. Emphysema was classified by LTRC repository centers using the patient’s clinical history, pulmonary function testing, CT findings evaluated by Board certified radiologists, and a reduction in diffusing capacity (DLCO). A final diagnosis of emphysema was made by the clinical center PI, who reviewed all available pathological and clinical participant information, as well as diagnosis reports from the tissue and radiology core laboratories. Tissue specimens from patients receiving a final diagnosis of moderate of very severe emphysema were compared to tissue from control smokers.

### Experimental emphysema

For elastase studies, anesthetized mice were intubated and given 0.25 U of porcine pancreatic elastase (Sigma) via 20G endotracheal catheter (Kent Scientific), as previously described^44^. For cigarette smoke exposure studies, we used the inhalation exposure apparatus (TE-10) by Teague Enterprises with 3R4F composition cigarettes (University of Kentucky Center for Tobacco Reference Products). Age matched mice beginning at 6–12 weeks of age were exposed to CS (~ 150 mg/m^3^) for a minimum of 3 h per day, 5 days a week^16^. For these studies, *C57Bl/6* or *Prox1-EGFP* reporter mice^21^ were used. Mice were housed in the Weill Cornell animal facility in 12/12 h light/dark cycles with ad libitum access to water and food. For all experiments, control and experimental animals were identically housed on the same rack in the animal facility. Both male and female mice were used in experimental and control groups. To assess for emphysema, we utilized morphometry software to quantify the length of chords within areas identified as airspace^45,46^. Using this method, it is possible to measure the size of the alveoli in all parts of the lung in a standardized and relatively automated manner^45,46^. Large airways, blood vessels, and other non-alveolar structures such as macrophages were manually removed from the images. Alveolar chord length for each image was calculated and the average mean chord length (MCL) for each mouse^46^.

### Functional assays of lymphatic drainage and leukocyte trafficking

Dextran drainage assays were performed as previously described^4^. 50 μl of 5 mg/ml dextran-555 (10,000 kDa MW, ThermoFisher) was administered to anesthetized mice intubated via 20G endotracheal catheter and fiberoptic light (Kent Scientific). Sixty minutes after administration, the mice were sacrificed for harvest of mediastinal lymph nodes, which were fixed in 4% PBS for 15 min prior to visualization. Lymph nodes were imaged using an Olympus SZX16 dissecting microscope. Quantification of fluorescence intensity was performed using ImageJ.

For cell trafficking experiments^4^, splenocytes were isolated from wild-type mouse spleens and cultured overnight with 100 ng/ml LPS and 5 μg/ml PHA (Sigma). The cells were then labeled with cell trace violet (CTV, Molecular Probes) according to manufacturer instructions. 1 × 10^7^ CTV-labeled cells were administered to anesthetized and intubated mice either via endotracheal catheter. Lymph nodes were harvested 48 h after administration of CTV-labeled cells. After digestion, single cell suspensions were blocked and then stained with FITC-conjugated anti-CD45 (eBioscience, 11-04551-82). Flow cytometry was performed using a BD FACSCanto and analyzed using FlowJo software to quantify CTV^+^ leukocytes in the lymph node cell suspensions.

### Whole mount staining

Whole mount staining of lung lymphatics from *Prox1-EGFP* mice was performed^4^. Mice were sacrificed and perfused with cold PBS. Lung tissue was harvested and fixed overnight in 4% PFA at 4 °C. Thick coronal sections were made using a scalpel under a fluorescent microscope to visualize and preserve the lymphatic vessels of the mainstem airways. Lung tissue was permeabilized in 0.1% BSA + 0.3% Triton-X in PBS overnight, then washed in PBS. The sections mounted in Vectashield (Vector) and imaged using a Leica TCS SP8 confocal microscope. Analysis and quantification of nuclear organization and roundness was performed using ImageJ.

### Immunohistochemistry

Mice were sacrificed and tissue was perfused with PBS. Prior to harvest, lungs were inflated with 4% PFA at constant pressure of 25 cmH_2_O. Lungs were fixed in 4% PFA overnight at 4 degrees. The tissue was then dehydrated and embedded in paraffin for sectioning. 6-µm sections were H&E stained or immunostained with antibodies for: VEGFR3 (R&D Systems, AF743) or CCL21 (R&D Systems, AF457), or Fibrinogen (Abcam, ab227063). Human lung tissue was stained with antibodies for PODOPLANIN (D240, Biolegend, 75782-960) and Fibrinogen (Abcam, ab227063) overnight at 4 degrees. After washing, slides were incubated with AlexaFluor-conjugated secondary antibodies for 2 h at room temperature. Slides were treated with Dapi-containing Vectashield and a cover slip was applied. Negative control slides were stained with secondary antibodies alone (Supplemental Fig. 5) to control for autofluorescence of lung tissue.

### Quantitative PCR

Lung tissue was harvested and homogenized for RNA isolation. Total RNA from lung tissue was isolated from lung tissue using RNEasy Kit (Qiagen). cDNA was made using Superscript III First-Strand Synthesis System (Invitrogen). cDNA was diluted 1:5 for qPCR reactions. Analysis of gene expression was performed using QuantStudio 6 Real-Time PCR System and SYBR Green PCR Master Mix (Applied Biosystems). Analysis of relative gene expression was carried out using the comparative CT method (ΔCT) using GAPDH as the reference housekeeping gene. Each qPCR reaction was performed in triplicate.

### Lymphatic permeability assays

To measure LEC permeability^47,48^, LECs were treated with cigarette smoke extract in the culture medium for the indicated amount of time. The underside of 1.0 µM pore size Transwell inserts (Falcon) were coated with 50 µg/ml collagen (Invitrogen). Then human primary LECs were plated at a density of 200,000 cells/cm^2^ on the underside. Models were cultured in EGM-2 (Lonza) at 37 °C and 5% CO2 for 48 h to ensure confluence. Transmural fluid flow of 1 µm/s using EGM-2 was introduced for 12 h to simulate the tissue microenvironment. Then, cigarette smoke extract (CSE) or DMEM (mock) was added to EGM-2 in the top well without added supplemental growth factors. Models were cultured in the new media under flow for two hours before flow was ceased, and 10 µg/ml fluorescently labeled 40 kDa and 150 kDa dextrans (Invitrogen) were added on top. The bottom well was assayed for fluorescence every hour for up to 12 h. Fluorescence intensity was measured using a plate reader (Tecan) and the amount of tracer transported was calculated using a standard curve. Effective permeability was estimated using the equation:

$${{P}}_{{{{eff}}}} = \frac{{{{C}}_{{{{lower}}}} {{V}}_{{{{lower}}}} }}{{{{tSC}}_{{{{initial}}}} }}$$

C = concentration, V_lower_ = volume of bottom compartment, S = surface area, T = time.

LEC monolayer was confirmed using immunofluorescence staining and trans endothelial and epithelial resistance (TEER, Millipore Sigma) was measured 12 h after CSE treatments.

### Immunofluorescence staining of LECs used for lymphatic transport model

Cells were fixed in 2% PFA for 15 min at room temperature and incubated with mouse anti-human VE-Cadherin (BD Sciences) at 4 °C overnight^48^. Secondary antibodies labeled with AlexaFluor 488 were used for detection. Slides were mounted using DAPI-containing Vectashield (Vector Laboratories) and imaged using a Zeiss Axio Observer. Image processing was performed using FIJI.

### Lymph harvest and proteomic analysis

Thoracic lymph was harvested from mice and samples were processed for proteomic analysis as previously described^49^ and detailed extensively in the online supplement. The mass spectrometry proteomics data have been deposited to the ProteomeXchange Consortium via the PRIDE^50^ partner repository with the dataset identifier PXD031413 and 10.6019/PXD031413.

### Statistics

Data are expressed as the mean ± SEM, and the numbers of samples per condition are indicated in the figure legends. Statistical significance was determined by unpaired, 2-tailed Student’s *t* test or ANOVA using GraphPad Prism software. P values of less than 0.05 were considered statistically significant. Quantification of lymphatics in lung tissue was performed using at least 5 randomly captured 10 × images of VEGFR3 staining per mouse. Lymphatic thrombosis was quantified as the number of VEGFR3^+^ lymphatic vessels with fibrin present in the lumen, as a percentage of total VEGFR3^+^ vessels in the image. For human samples, number of lymphatics was calculated as the number of PDPN^+^ vessels per 10 × image. At least 3 randomly captured 10 × images were used per sample. Lymphatic thrombosis was calculated as PDPN^+^ lymphatic vessels with fibrin in the lumen, expressed as a percentage of total PDPN^+^ vessels. TLOs were defined as discrete lymphocyte-dense accumulations on H&E-stained sections. Number of TLOs was quantified using 10 × images representing the entirety of the lung tissue section for each patient sample.

### Study approval

All animal experiments were approved by the IACUC of Weill Cornell Medicine and performed in accordance with relevant guidelines and regulations. Reporting in the manuscript follows the recommendations in the ARRIVE guidelines.

## Supplementary Information

Supplementary Information.

Supplementary Table 1.

## References

[1] Clement CC, Rotzschke O, Santambrogio L (2011). The lymph as a pool of self-antigens. Trends Immunol.

[2] Clement CC (2018). Quantitative profiling of the lymph node clearance capacity. Sci. Rep..

[3] El-Chemaly S, Levine SJ, Moss J (2008). Lymphatics in lung disease. Ann. N. Y. Acad. Sci..

[4] Reed HO (2019). Lymphatic impairment leads to pulmonary tertiary lymphoid organ formation and alveolar damage. J. Clin. Investig..

[5] Aloisi F, Pujol-Borrell R (2006). Lymphoid neogenesis in chronic inflammatory diseases. Nat. Rev. Immunol..

[6] Hogg JC (2004). The nature of small-airway obstruction in chronic obstructive pulmonary disease. N. Engl. J. Med..

[7] Mori M (2013). Appearance of remodelled and dendritic cell-rich alveolar-lymphoid interfaces provides a structural basis for increased alveolar antigen uptake in chronic obstructive pulmonary disease. Thorax.

[8] Baluk P (2014). Preferential lymphatic growth in bronchus-associated lymphoid tissue in sustained lung inflammation. Am. J. Pathol..

[9] Rangel-Moreno J (2006). Inducible bronchus-associated lymphoid tissue (iBALT) in patients with pulmonary complications of rheumatoid arthritis. J. Clin. Investig..

[10] Sato M (2009). The role of intrapulmonary de novo lymphoid tissue in obliterative bronchiolitis after lung transplantation. J. Immunol..

[11] Yadava K, Bollyky P, Lawson MA (2016). The formation and function of tertiary lymphoid follicles in chronic pulmonary inflammation. Immunology.

[12] Hardavella G (2012). Lymphangiogenesis in COPD: Another link in the pathogenesis of the disease. Respir. Med..

[13] Mori M, Andersson CK, Graham GJ, Lofdahl CG, Erjefalt JS (2013). Increased number and altered phenotype of lymphatic vessels in peripheral lung compartments of patients with COPD. Respir. Res..

[14] Baluk P, McDonald DM (2018). Imaging lymphatics in mouse lungs. Methods Mol. Biol..

[15] Baluk P, McDonald DM (2008). Markers for microscopic imaging of lymphangiogenesis and angiogenesis. Ann. N. Y. Acad. Sci..

[16] Cloonan SM (2016). Mitochondrial iron chelation ameliorates cigarette smoke-induced bronchitis and emphysema in mice. Nat. Med..

[17] van der Strate BW (2006). Cigarette smoke-induced emphysema: A role for the B cell?. Am. J. Respir. Crit. Care Med..

[18] John-Schuster G (2014). Cigarette smoke-induced iBALT mediates macrophage activation in a B cell-dependent manner in COPD. Am. J. Physiol. Lung Cell. Mol. Physiol..

[19] Suki B, Bartolak-Suki E, Rocco PRM (2017). Elastase-induced lung emphysema models in mice. Methods Mol. Biol..

[20] Yang Y, Cha B, Motawe ZY, Srinivasan RS, Scallan JP (2019). VE-cadherin is required for lymphatic valve formation and maintenance. Cell Rep..

[21] Choi I (2011). Visualization of lymphatic vessels by Prox1-promoter directed GFP reporter in a bacterial artificial chromosome-based transgenic mouse. Blood.

[22] Cook DN, Bottomly K (2007). Innate immune control of pulmonary dendritic cell trafficking. Proc. Am. Thorac. Soc..

[23] Jakubzick C, Tacke F, Llodra J, van Rooijen N, Randolph GJ (2006). Modulation of dendritic cell trafficking to and from the airways. J. Immunol..

[24] Johnson LA, Jackson DG (2014). Control of dendritic cell trafficking in lymphatics by chemokines. Angiogenesis.

[25] Russo E (2016). Intralymphatic CCL21 promotes tissue egress of dendritic cells through afferent lymphatic vessels. Cell Rep..

[26] Nakano H, Gunn MD (2001). Gene duplications at the chemokine locus on mouse chromosome 4: Multiple strain-specific haplotypes and the deletion of secondary lymphoid-organ chemokine and EBI-1 ligand chemokine genes in the plt mutation. J. Immunol..

[27] Hansen KC, D'Alessandro A, Clement CC, Santambrogio L (2015). Lymph formation, composition and circulation: A proteomics perspective. Int. Immunol..

[28] Clement CC, Santambrogio L (2013). The lymph self-antigen repertoire. Front. Immunol..

[29] Baluk P (2007). Functionally specialized junctions between endothelial cells of lymphatic vessels. J. Exp. Med..

[30] Zhang F, Zarkada G, Yi S, Eichmann A (2020). Lymphatic endothelial cell junctions: Molecular regulation in physiology and diseases. Front. Physiol..

[31] Demoor T, Bracke KR, Joos GF, Brusselle GG (2010). Increased T-regulatory cells in lungs and draining lymph nodes in a murine model of COPD. Eur. Respir. J..

[32] Demoor T (2009). CCR7 modulates pulmonary and lymph node inflammatory responses in cigarette smoke-exposed mice. J. Immunol..

[33] Fader RC, Ewert A (1986). Evolution of lymph thrombi in experimental *Brugia malayi* infections: A scanning electron microscopic study. Lymphology.

[34] Kochilas LK, Shepard CW, Berry JM, Chin AJ (2014). Ultrasonographic imaging of the cervical thoracic duct in children with congenital or acquired heart disease. Echocardiography.

[35] Lippi G, Favaloro EJ, Cervellin G (2012). Hemostatic properties of the lymph: Relationships with occlusion and thrombosis. Semin. Thromb. Hemost..

[36] Marcus RT, Pawade J, Vella EJ (1990). Painful lymphatic occlusion following axillary lymph node surgery. Br. J. Surg..

[37] Dumont AE, Mulholland JH (1960). Flow rate and composition of thoracic-duct lymph in patients with cirrhosis. N. Engl. J. Med..

[38] Messner B, Bernhard D (2014). Smoking and cardiovascular disease: Mechanisms of endothelial dysfunction and early atherogenesis. Arterioscler. Thromb. Vasc. Biol..

[39] Lu Q, Gottlieb E, Rounds S (2018). Effects of cigarette smoke on pulmonary endothelial cells. Am. J. Physiol. Lung Cell. Mol. Physiol..

[40] Tapson VF (2005). The role of smoking in coagulation and thromboembolism in chronic obstructive pulmonary disease. Proc. Am. Thorac. Soc..

[41] Coughlin SR (2005). Protease-activated receptors in hemostasis, thrombosis and vascular biology. J. Thromb. Haemost..

[42] Martorell L (2008). Thrombin and protease-activated receptors (PARs) in atherothrombosis. Thromb. Haemost..

[43] Minami T (2004). Thrombin and phenotypic modulation of the endothelium. Arterioscler. Thromb. Vasc. Biol..

[44] Hisata S (2021). Reversal of emphysema by restoration of pulmonary endothelial cells. J. Exp. Med..

[45] Laucho-Contreras ME, Taylor KL, Mahadeva R, Boukedes SS, Owen CA (2015). Automated measurement of pulmonary emphysema and small airway remodeling in cigarette smoke-exposed mice. J. Vis. Exp..

[46] Cloonan SM, Choi AM (2016). Mitochondria in lung disease. J. Clin. Investig..

[47] Chen ZH (2010). Autophagy protein microtubule-associated protein 1 light chain-3B (LC3B) activates extrinsic apoptosis during cigarette smoke-induced emphysema. Proc. Natl. Acad. Sci. U.S.A..

[48] Triacca V, Guc E, Kilarski WW, Pisano M, Swartz MA (2017). Transcellular pathways in lymphatic endothelial cells regulate changes in solute transport by fluid stress. Circ. Res..

[49] Zawieja DC (2019). Lymphatic cannulation for lymph sampling and molecular delivery. J. Immunol..

[50] Perez-Riverol Y (2022). The PRIDE database resources in 2022: A hub for mass spectrometry-based proteomics evidences. Nucleic Acids Res..

