# Supplementary material for: Lung lymphatic thrombosis and dysfunction caused by cigarette smoke exposure precedes emphysema in mice
Source: Sci Rep. 2022 Mar 23;12:5012. doi: 10.1038/s41598-022-08617-y (PMC8943143; doi:10.1038/s41598-022-08617-y)
Supplement: Supplementary file 1 — Supplementary Information. [file 41598_2022_8617_MOESM1_ESM.docx]

Lung Lymphatic Thrombosis and Dysfunction Caused by Cigarette Smoke Exposure Precedes Emphysema in Mice

Barbara D. Summers^1^, Kihwan Kim^1^, Cristina C. Clement^2^, Zohaib Khan^2^, Sangeetha Thangaswamy^2^, Jacob McCright^3^, Katharina Maisel^3^, Sofia Zamora^1^, Stephanie Quintero^1^, Alexandra C. Racanelli^1^, David Redmond^4^, Jeanine D’Armiento^5^, Jisheng Yang^6^, Amy Kuang^1^, Laurel Monticelli^1^, Mark L. Kahn^6^, Augustine M. K. Choi^1^, Laura Santambrogio^2^, and Hasina Outtz Reed^1*^

Supplemental Data and Methods

**Supplemental Figure 1. Lymphatic thrombosis associated with tertiary lymphoid organs (TLOs) in CS-exposed mice.** (A) Quantification of mean cord length (MCL) does not demonstrate emphysema after 4 months of CS exposure in mice. (B) Quantification of TLOs after 4 months of CS exposure compared to room air. (C,D, F,G) H&E of lung tissue after 4 months of CS exposure or room air. TLO indicated with arrowhead (F) and higher magnification of the same area with asterisk (G). (H) Serial section immunohistochemical staining of TLO seen in (G) for VEGFR3 (red) and fibrinogen (green). Thrombosed lymphatic indicated with arrowhead adjacent to TLO (asterisk). Serial section of lung tissue from room air control mouse shown in D,E. At least 5 independent 10x images of lung tissue from each mouse were used for quantification of TLOs. MCL was quantified using at least 5 20X images per mouse. All values expressed as mean ± SEM. *P* value calculated by Student’s *t* test. ns = not significant. Scale bars = 50µm.

**Supplemental Figure 2: Elastase-induced emphysema does not lead to lung lymphatic thrombosis.** (A) Quantification of VEGFR3^+^ lymphatics in lung tissue from mice 21 days after intratracheal porcine pancreatic elastase (PPE) or PBS as a control. (B) Quantification of lymphatic thrombosis, as expressed by percentage of VEGFR3^+^ vessels with luminal fibrin, after PPE or PBS. (C,D) Immunohistochemistry for VEGFR3 (red) and fibrinogen (green) in lung tissue from mice treated with PPE or PBS. (E-H) H&E staining of lung tissue from PPE-treated or PBS-treated mice. At least 5 independent 10x images of lung tissue were used for each mouse tissue sample for quantification. All values expressed as mean ± SEM. *P* value calculated by Student’s *t* test. ns = not significant. Scale bars = 25µm.

**Supplemental Figure 3: Increased complement in lymph from CS-exposed mice.** (A) Relative abundance of complement proteins in lymph from CS-exposed or room air control mice, as quantified by normalized average MS1+MS2 intensities by mass spectrometry and DIA proteomics. (B) View of IPA-predicted complement system in lymph from CS-exposed mice compared to room air control mice. The log ratio of proteins in lymph from CS-exposed versus room air mice are indicated. Proteins that were upregulated are depicted in red, and those that were downregulated are shown in green. Proteins that were predicted by IPA to be inhibited due to their networking relationship with the experimentally determined proteins are shown in blue, and proteins that were predicted to be activated are shown orange. Results from 2 independent experiments with a total of n = 10 CS-exposed mice and n = 12 room air mice. Results are reported as mean ± SDV and the statistical significance corresponding to p<0.05 was calculated in GraphPad Prism 9 (GraphPad Software, La Jolla, CA) using the multiple nonparametric Mann-Whitney t-tests discovery analysis with Holm-Sidak method (alpha=0.05).

**Supplemental Figure 4: No change in the expression of junctional proteins with cigarette smoke extract (CSE) exposure.** (A) Quantitative PCR for VE-cadherin using cDNA from control LECs or LECs exposed to 2% CSE for 24 hours, normalized to GAPDH. (B,C) Western blots for VE-Cadherin, PECAM, and occludin using lysates from control LECs or LECs exposed to 2% CSE for 24 hours. GADPH was probed as a loading control.

**Supplemental Figure 5: Negative controls for lung tissue staining.** (A-C) Serial sections of mouse lung tissue stained with (A) CCL21 (B) VEGFR3 (C) Fibrinogen. Left panel is adjacent section with secondary antibody alone, right panel was stained with the indicated primary and secondary antibodies. (D,E) Serial sections of human lung tissue stained with fibrinogen (D) and Podoplanin (D2-40). Left panel is adjacent section with secondary antibody alone, right panel was stained with the indicated primary and secondary antibodies. Scale bars = 25µm.

**Supplemental Methods**

Lymph Harvest and Proteomic Analysis

*Chemicals and other reagents*

Acetic acid (ULC/MS grade), acetonitrile, formic acid, methanol, trifluoroacetic acid (TFA), and ULC/MS-grade water (for nano-LC analysis, 99% purity) were purchased from Fisher Scientific. TCEP-HCl, iodoacetamide, ammonium bicarbonate, glycine, urea, thiourea, KCl, KH_2_PO_4_, H_3_PO_4_, and Na_2_CO_3_ were of the highest grade available from Sigma-Aldrich Millipore. Porcine trypsin (20 mg, specific activity >5,000 units/mg sequencing grade modified), Lys-C (sequencing grade, 10  mg) and Glu-C, sequencing grade (10 mg) were purchased from Promega (Madison, WI). All solutions were prepared using MilliQ water purified by an Elix 3 UV Water Purification System (Millipore, Billerica, USA) and filtered through 0.2 um pore membrane sterile filter units (Steritop^TM^, Millipore). All methods were performed in accordance with the relevant guidelines and regulations. Total protein quantitation was performed using the Micro BCA™ Protein Assay Kit, (cat # 23235 from Thermofisher Scientific). Amicon Ultra-0.5 ml centrifugal filters (Ultracel-10K, cat#UFC501024) were purchased from Millipore-Sigma.

*Lymph samples from mice exposed to CS or room air*

Both male and female mice were anesthetized with alpha chloralose (2%) and urethane (10%) and kept in supine position in the dissection board. The chest was opened and washed with 1XPBS to remove any blood. The thoracic duct vessel close the aorta was located removed from the surrounding fat tissue using fine forceps (Dumont #5 tip size 0.25 X 0.05 mm tip size) without damaging the thoracic duct vessel. A glass cannula attached to a 1ml syringe was inserted into the upper part of the thoracic duct and collect the lymph fluid from thoracic duct. Once collected, lymph fluid was immediately transferred into Eppendorf tubes and spin down to remove the immune cells and collect the supernatant, filtered (0.22um) and further added 1X protease inhibitor cocktail and stored at -80C for further processing.

*Extraction of endogenous peptides (peptidome) from lymph sample*

Equal amounts of 50-100 ug of total protein from the lymph collected from mice exposed to “room air” vs “CS”, (n=12 biological replicates for room air and n=10 for smoke air) were equilibrated in 0.4 ml of sterile PBS buffer supplemented with a cocktail of protease inhibitors (Roche). Peptides were extracted using 0.2% TFA. Samples were then filtrated through a 10,000-Da cutoff filter device (Amicon) at 10°C for 30 minutes, desalted using pepClean C-18 spin columns (Pierce), and eluted with 70% acetonitrile containing 0.1% TFA for further nanoLC/MS/MS analysis.

*Processing of lymph samples for proteomics analysis*

50-100 μg from each lymph sample (n=22 biological replicates for each sample category, i.e., “room air” and “CS” conditions) were reduced with 25 mM TCEP-HCl (Thermo Scientiﬁc) in 50 mM ammonium bicarbonate (ABC) buffer, containing 8 M urea at pH 8.5 for 45 minutes at RT followed by alkylation with 100 mM iodoacetamide for 50 minutes in the dark at RT. The protein solutions were transferred on microcon-10kDa centrifugal filter units with Ultracel-10 membrane (catalog# MRCPRT010) from Millipore Sigma and washed with 50 mM ammonium bicarbonate buffer five times at 9000xg in a microcentrifuge, for 10 minutes each step. The reduced and alkylated samples were resuspended in 100 μl of 50 mM ABC buffer, pH 8.9 (urea <2M) and digestion was carried out at 37°C overnight (12 hours) using a combination of three enzymes: trypsin/LysC at 20:1 protein: enzyme ratio and GluC at 10:1 protein: enzyme ratio. The digestion was quenched with 0.5% acetonitrile and 1.5% formic acid. Processed peptides were then extracted through a 10-kDa MWCO (molecular weight cut-off) using 10kDa centrifugal filter units by spinning at 10,000xg for 15 minutes in a 20:1 microcentrifuge. The final peptide mixture, extracted from all enzymatic digestions, was desalted on C18 Prep clean columns (EMD Millipore) and reconstituted in 25 µl 5% acetonitrile containing 0.1% (v/v) trifluoroacetic acid for further nanoLC/MS/MS analysis.

Equal aliquots (ug) of endogenous peptides (MW<10 kDa) and/or tryptic peptides were analyzed in replicates, (n=12 biological replicates for room air and n=10 for smoke air) by nano-LC-MS/MS using a combination of data dependent and independent analyses (DDA and DIA, respectively). We used a Thermo Scientific™ Orbitrap Fusion™ Tribrid™ mass spectrometer and applied a protocol developed and published previously (Clement CC et al., Immunity 2021). Briefly, desalted peptides were injected onto an EASY-Spray PepMap RSLC C18 50 cm x 75 μm column (Thermo Scientific), which was coupled to the mass spectrometer. Peptides were eluted with a non-linear 180 min gradient of 5-30% buffer B (0.1% (v/v) formic acid, 100% acetonitrile) at a flow rate of 250 nL/min. The column temperature was maintained at a constant 50 ˚C during all experiments. For DIA analysis, survey scans of peptide precursors were performed from 350-1200 *m/z* at 120K FWHM resolution (at 200 *m/z*) with a 1 x 10^6^ ion count target and a maximum injection time of 60 ms. The instrument was set to run in top speed mode with 3 s cycles for the survey and the MS/MS scans. After a survey scan, 26 m/z DIA segments were acquired from 200-2000 *m/z* at 60K FWHM resolution (at 200 *m/z*) with a 1 x 10^6^ ion count target and a maximum injection time of 118 ms. HCD fragmentation was applied with 27% collision energy and resulting fragments were detected using the rapid scan rate in the Orbitrap. The spectra were recorded in profile mode.

*DDA nano-LC/MS/MS for generation of spectral libraries*

The sample specific spectral library (SSL) was generated by pooling 1/10 aliquots from each biological sample and DDA method for peptide MS/MS analysis. Survey scans of peptide precursors were performed from 400 -1500 *m/z* at 120K FWHM resolution (at 200 *m/z*) with a 4 x 10^5^ ion count target and a maximum injection time of 50 ms. The instrument was set to run in top speed mode with 3 s cycles for the survey and the MS/MS scans. After a survey scan, tandem MS was performed on the most abundant precursors exhibiting a charge state from 2 to 6 of greater than 5 x 10^3^ intensity by isolating them in the quadrupole at 1.6 Th. CID fragmentation was applied with 35% collision energy and resulting fragments were detected using the rapid scan rate in the ion trap. The AGC target for MS/MS was set to 1 x 10^4^ and the maximum injection time limited to 35 ms. The dynamic exclusion was set to 60 s with a 10-ppm mass tolerance around the precursor and its isotopes. Monoisotopic precursor selection was enabled. The remaining half of each sample was run using DIA method as described above.

Data Processing Protocol

*Generation of Spectral Libraries*

To generate the spectral libraries, the acquired DDA raw files corresponding to the pooled samples from the biological replicates (aliquots of 1/10 from each biological replicate for each “room air” and “CS” lymph sample set) were searched with PEAKS X+ and then filtered with Scaffold software (version 4.6.2). The enzyme restriction was set up as “*no enzyme*” option in PEAKS X+ to fit the endogenously processed peptides. Then, the spectral library was exported as a *“.blib”* file using the built-in available option from the Scaffold software.

An independent analysis of the MS/MS DDA raw files was performed using the MSFragger (The Nesvizhskii Lab, 1301 Catherine, 4237 Medical Science I, Ann Arbor, MI 48109: version 3.2). MSFragger was set up to search a reverse concatenated uniprot-filtered-organism Mus musculus (mouse) database (April 2021, 36,902 entries) assuming the digestion enzyme trypsin for proteomics samples. MSFragger was searched with a fragment ion mass tolerance of 20 PPM and a parent ion tolerance of 20 PPM. Deamidated of asparagine and glutamine, oxidation of histidine, methionine, and tryptophan and carbamidomethyl of cysteine were specified in MSFragger as fixed modifications. Scaffold (version Scaffold_5.0.1, Proteome Software Inc., Portland, OR) was used to validate MS/MS based peptide and protein identifications. Peptide identifications were accepted if they could be established at greater than 95.0% probability by the Scaffold Local FDR algorithm. Protein identifications were accepted if they could be established at greater than 95.0% probability and contained at least 1 identified peptide. Protein probabilities were assigned by the Protein Prophet algorithm (Nesvizhskii, Al et al Anal. Chem. 2003;75(17):4646-58). Proteins that contained similar peptides and could not be differentiated based on MS/MS analysis alone were grouped to satisfy the principles of parsimony. Then, the spectral library was exported as a “.blib” file using the built-in available option from the Scaffold software.

*DIA Analysis of peptidomes and proteomics data*

DIA data were analyzed using Scaffold DIA (1.2.1) (Proteome Software Inc., Portland) which had the raw data files converted to mzML format using ProteoWizard (3.0.11748). The analytic samples were aligned based on retention times and individually searched against “DDA.blib spectral library” with a peptide mass tolerance of 10 to 15 ppm and a fragment mass tolerance of 15 to 50.0 ppm. Variable modifications were imported from the DDA based spectral library as follow: methionine, lysine, proline, arginine, cysteine, and asparagine oxidations (+15.99 on CKMNPR), deamidation of asparagine and glutamine (NQ-0.98) and pyro-Glu from glutamine (Q-18.01 N-term). The *“no enzyme*” option was used in Scaffold DIA with variable allowed 8-12 missed cleavage site(s) for peptidomics analysis. Only peptides with charges in the range [2-8] and length in the range [5-25] were exported for further quantitation. For the proteomics analysis, “trypsin” restriction was used for the enzyme digestion and one “allowed missed cleavage” Peptides identified in each sample were filtered by Percolator (3.01. nightly-13-655e4c7-dirty) to achieve a maximum FDR between 0.01-0.05. Individual search results were combined, and peptide identifications were assigned posterior error probabilities and re-filtered to FDR thresholds of 0.01-0.05 by Percolator (3.01. nightly-13-655e4c7-dirty). Peptide quantification was performed by Encyclopedia (0.7.2). For each peptide, the 5 highest quality fragment ions were selected for quantitation. The intensities for the proteins were calculated and normalized by summation of the peptide intensities using the Scaffold DIA’s built-in normalization algorithm.

*Independent analysis of DIA data files from peptidomes and proteomics with PEAKS X+/pro*

Raw files from each biological replicate corresponding to the lymph “room air” and “CS” samples were filtered using the DIA built-in option in PEAKS X+ /Pro(Bioinformatics Solutions, Waterloo, Canada), de novo sequenced, and assigned with protein ID using by searching against the mouse Swiss-Prot database (April 2021; 36,902 entries) and the following search parameters trypsin/LysC/Glu-C, as restriction enzymes in the case of proteomics data set and “*no enzyme*” for searching the peptidomics dataset; two allowed missed cleaves at one peptide end was applied for tryptic peptides. The parent mass tolerance was set to 13 ppm using monoisotopic mass, and fragment ion mass tolerance was set to 0.03 Da. Carbamidomethyl cysteine (+57.0215 on C) was specified as a fixed modification. Methionine, lysine, proline, arginine, cysteine, and asparagine oxidations (+15.99 on CKMNPR), deamidation of asparagine and glutamine (NQ-0.98), and pyro-Glu from glutamine (Q-18.01 N-term) were set as variable modifications. We also performed an additional analysis of peptidomes and proteomics DIA files using the “*Spectral Libraries*” search option enabled by the PEAKS X/Pro, using the spectral libraries generated from DDA raw files as described above. Data were validated using the false discovery rate (FDR) method built in PEAKS X+, and protein identifications were accepted with a confidence score (−10lgP) >15 for peptides and (−10lgP) >15 for proteins; a minimum of one peptide per protein was allowed after data were filtered for <5% FDR for endogenous peptides and FDR<1% for tryptic peptides; and <3% FDR for proteins identifications (P < 0.05).

*Statistical analysis of label free quantitation (LFQ) analyses*

Statistical analysis of LFQ was performed on average values from normalized MS1 areas and MS2 intensities from DIA data for each biological replicate, using Windows GraphPad Prism 8 and higher (GraphPad Software, La Jolla, CA) (n=2 independent experiments for each biological replicate). Numerical results are reported as mean ± SE or ±SDV when appropriate. Data are derived from a minimum of two independent experiments unless stated otherwise. Multiple nonparametric Mann-Whitney t-tests discovery analysis with Holm-Sidak method (p<0.05 for statistical significance) was performed for the experiments aimed to compare the changes in the protein expression profiles across many proteins within one pathway, such as across coagulation, prothrombin activation and complement pathways. Statistical significance is shown as *, P < 0.05; **, P < 0.01; ***, P < 0.001.

*Gene ontology, molecular, and cellular pathways enrichment analysis*

Networks, functional analyses, biochemical, and cellular pathways were generated using IPA (Ingenuity Systems) using the proteins identified from proteomics and LFQ analyses (Supplementary Table 1). Specifically, the experimentally determined protein ratios corresponding to [Smoke/Room Air] from MS1 And MS2 analysis of DIA proteomics data were used to calculate the experimental fold changes by rescaling their values using a log2 transformation, such that positive values reflected fold increases while the negative values reflected fold decreases. For network generation, datasets containing gene identifiers (gene symbols) for the [Smoke/Room Air] were uploaded into the IPA application together with their rescaled log2 transformation of the protein’s average ratios from n=12 and n=10 biological lymph replicates from mice exposed to room and smoke air, respectively. These molecules were overlaid onto a global molecular network contained in the Ingenuity Knowledge Base. The networks were then algorithmically generated based on their connectivity index using the built-in IPA algorithm. The probability of having a relationship between each IPA indexed biological function and the experimentally determined genes was calculated by a right-tailed Fisher’s exact test. The level of significance was set to P < 0.05. Accordingly, the IPA analysis identified the molecular and cellular pathways from the IPA library of canonical pathways that were most significant to the dataset (−log (P value) > 1.3). For the quantitative analysis of the expression profiles, IPA assigned the z-score function to all eligible canonical and cellular pathways (where z < −2 represents significant down-regulation while z > 2.0 represents a significant up-regulation of the selected pathways, see Supplementary Table 1 for details).

The mass spectrometry proteomics data have been deposited to the ProteomeXchange Consortium via the PRIDE^1^ partner repository with the dataset identifier PXD031413 and 10.6019/PXD031413.

1 Perez-Riverol, Y. *et al.* The PRIDE database resources in 2022: a hub for mass spectrometry-based proteomics evidences. *Nucleic Acids Res* **50**, D543-D552, doi:10.1093/nar/gkab1038 (2022).
